# Models of chronic obstructive pulmonary disease

**DOI:** 10.1186/1465-9921-5-18

**Published:** 2004-11-02

**Authors:** David A Groneberg, K Fan Chung

**Affiliations:** 1 Pneumology and Immunology, Otto-Heubner-Centre, Charité School of Medicine, Free University and Humboldt-University, Berlin, Germany; 2 Thoracic Medicine, National Heart & Lung Institute, Imperial College, London, UK

**Keywords:** Chronic obstructive pulmonary disease, COPD, asthma, animal, mice, rat, guinea pig, tobacco smoke, nitrogen dioxide, sulfur dioxide

## Abstract

Chronic obstructive pulmonary disease (COPD) is a major global health problem and is predicted to become the third most common cause of death by 2020. Apart from the important preventive steps of smoking cessation, there are no other specific treatments for COPD that are as effective in reversing the condition, and therefore there is a need to understand the pathophysiological mechanisms that could lead to new therapeutic strategies. The development of experimental models will help to dissect these mechanisms at the cellular and molecular level. COPD is a disease characterized by progressive airflow obstruction of the peripheral airways, associated with lung inflammation, emphysema and mucus hypersecretion. Different approaches to mimic COPD have been developed but are limited in comparison to models of allergic asthma. COPD models usually do not mimic the major features of human COPD and are commonly based on the induction of COPD-like lesions in the lungs and airways using noxious inhalants such as tobacco smoke, nitrogen dioxide, or sulfur dioxide. Depending on the duration and intensity of exposure, these noxious stimuli induce signs of chronic inflammation and airway remodelling. Emphysema can be achieved by combining such exposure with instillation of tissue-degrading enzymes. Other approaches are based on genetically-targeted mice which develop COPD-like lesions with emphysema, and such mice provide deep insights into pathophysiological mechanisms. Future approaches should aim to mimic irreversible airflow obstruction, associated with cough and sputum production, with the possibility of inducing exacerbations.

## Introduction

The global burden of disease studies point to an alarming increase in the prevalence of chronic obstructive pulmonary disease (COPD) [1] which is predicted to be one of the major global causes of disability and death in the next decade [2]. COPD is characterized by a range of pathologies from chronic inflammation to tissue proteolysis and there are no drugs specifically developed for COPD so far. Cessation of cigarette smoking is accompanied by a reduction in decline in lung function [3] and is a most important aspect of COPD management. The mainstay medication consists of beta-adrenergic and anticholinergic bronchodilators; addition of topical corticosteroid therapy in patients with more severe COPD provides may enhance bronchodilator responses and reduce exacerbations [4].

**Figure 1:**
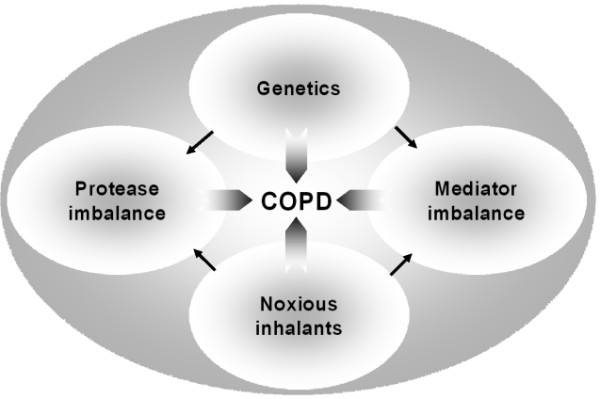
**Potential pathogenetic mechanisms involved in COPD **Exogenous inhaled noxious stimuli such as tobacco smoke, noxious gases or indoor air pollution and genetic factors are proposed to be the major factors related to the pathogenesis of COPD. These factors may influence protease activity and may also lead to an imbalance between pro-inflammatory and anti-inflammatory mediators.

In contrast to the large amount of experimental studies on allergic asthma and the detailed knowledge that exists on mediators of allergic airway inflammation [5,6], much less has been conducted for COPD. More effort and resources have been directed into asthma research in comparison to COPD. The available insights into the pathogenesis and pathophysiology of asthma may help to improve research in COPD [7]. Many research centres that previously focused on asthma now also investigate mechanisms of COPD. Using molecular and genetic approaches, an increasing range of molecules has been identified that could underlie the pathogenic inflammation of chronic allergic airway inflammation [8]. Based on these findings and on new ways of administering drugs to the lungs [9], a new image of overwhelming complexity of the underlying pathophysiology of COPD has emerged (Figure 1). The current challenge in COPD research is to identify the role of the various mediators and molecular mechanisms that may be involved in its pathophysiology, and obtain new treatments. In addition, it is incumbent to understand the effect of smoking cessation on the pathogenetic process.

Studying the molecular pathways in human subjects is restricted to the use of morphological and molecular assessment of lung tissues obtained at surgery or performing limited in vitro studies at one single point in time [10]. There is a need for *in vivo *animal models to examine more closely pathogenesis, functional changes and the effects of new compounds or treatments. However, animal models have limitations since there is no spontaneous model, and models do not necessarily mimic the entire COPD phenotype. The best model remains chronic exposure to cigarette smoke, since this is the environmental toxic substance(s) that cause COPD in man. However, other substances are also implicated such as environmental pollution due to car exhaust fumes. The present review draws attention to specific aspects of functional and structural features of COPD that need to be realized when interpreting molecular mechanisms identified in animal models of COPD. It identifies important issues related to the ongoing experimental COPD research which may in the future provide optimized COPD diagnosis and treatment.

### COPD

#### Clinical features

Before characterizing and discussing the different animal models of COPD which have been established so far, it is crucial to reflect that within COPD, different disease stages exist and that only some of them may be mimicked in animal models. The diagnosis of COPD largely relies on a history of exposure to noxious stimuli (mainly tobacco smoke) and abnormal lung function tests. Since COPD has a variable pathology and the molecular mechanisms are only understood to a minor extent, a simple disease definition has been difficult to establish. However, the diagnosis of COPD relies on the presence of persistent airflow obstruction in a cigarette smoker [4].

A classification of disease severity into four stages has been proposed by the GOLD guidelines based primarily on FEV1 [4]. The staging on the basis of FEV1 alone as an index of severity for COPD has been criticised. A composite measure essentially based on clinical parameters (BODE) has been shown to be better at predicting mortality than FEV1 [11]. The natural history of COPD in terms of evolution of FEV1 remains unclear and the temptation is to regard the stages as evolving from Stage 0 to Stage 4. Just as many smokers do not develop COPD, it is possible that the disease may not progress from one stage to the next. Some patients with severe COPD are relatively young and it is not clear if early stages of their disease are similar to those found in patients with mild COPD. COPD is a heterogeneous disease and different possible outcomes may occur at each of the stages. Experimental modeling of each stage of severity may be a way of providing an answer to this issue. Animal models may also help to provide a better classification of severity by correlating biochemical, molecular and structural changes with lung function and exercise tolerance.

#### Pathophysiology

The presence of airflow obstruction which has a small reversible component, but which is largely irreversible is a major feature of COPD as indicated by the Global Initiative for Chronic Obstructive Lung Disease (GOLD) guidelines [4]. It is proposed to be the result of a combination of small airways narrowing, airway wall inflammation [12] and emphysema-related loss of lung elastic recoil [13,14]. These features differ to a large extent to findings observed in bronchial asthma (Table 1) where airflow obstruction is usually central, while involvement of the small airways occurs in more severe disease. The degree of airflow obstruction in COPD can be variable, but loss of lung function over time is a characteristic feature. Ideally, the development of airflow obstruction which is largely irreversible but has a small reversible component should be a feature of animal models of COPD, but this has not been reproduced so far. One of the important limitations of animal models of COPD is the difficulty in: reproducing small airways pathology particularly when working in small animals, particularly the mouse and rat where there are few levels of airway branching. This is a problem inherent to small laboratory animal models but provides an advantage for developing models in larger animals such as the pig or sheep. Part of the problem of analyzing small airways is also due to the lack of sophistication of lung function measurements, particularly in mice, but there has been recent development in the methodology of lung function measurement [15]. A new ex-vivo method of analyzing the airway periphery is by the technique of precision cut lung slices combined to videomorphometry [16,17].

**Table 1:** Currently known phenotype differences between COPD and asthma

**Feature**	**COPD**	**Asthma**
**Limitation of Airflow**	Largely irreversible	Largely reversible
**Parenchymal integrity**	destruction	intact
**Bronchial Hyperresponsiveness**	Variable (small)	significant
**Steroid response**	reduced or absent	present

In addition to pulmonary alterations, other organ systems may be affected in COPD [18]. Systemic effects of COPD include weight loss, nutritional abnormalities and musculoskeletal dysfunction. These systemic manifestations will gain further socioeconomic importance with an increasing prevalence of COPD in the next years [19]. Therefore, these systemic effects should be present in animal models of COPD and further analysis of mechanisms underlying these systemic effects in experimental models may help to optimize disease management.

#### Inflammatory cells

An important feature of COPD is the ongoing chronic inflammatory process in the airways as indicated by the current GOLD definition of COPD [4]. There are differences between COPD and asthma: while mast cells and eosinophils are the prominent cell types in allergic asthma, the major inflammatory cell types in COPD are different (Table 2) [20-22].

**Table 2:** Differences in inflammatory cells between COPD and asthma. Ranked in relative order of importance.

**COPD**	**Asthma**
Neutrophils	Eosinophils
Macrophages	Mast cells
CD8-T-lymphocytes	CD4-T-lymphocytes
Eosinophils (exacerbations)	Macrophages, Neutrophils

Neutrophils play a prominent role in the pathophysiology of COPD as they release a multitude of mediators and tissue-degrading enzymes such as elastases which can orchestrate tissue destruction and chronic inflammation [8,23]. Neutrophils and macrophages are increased in bronchoalveolar lavage fluid from cigarette smokers [24]. Patients with a high degree of airflow limitation have a greater induced sputum neutrophilia than subjects without airflow limitation. Increased sputum neutrophilia is also related to an accelerated decrease in FEV_1 _and sputum neutrophilia is more prevalent in subjects with chronic cough and sputum production [25].

The second major cell type involved in cellular mechanisms are macrophages [26]. They can release numerous tissue-degrading enzymes such as matrix metalloproteinases (MMPs). In an animal model of tobacco smoke-induced tissue matrix degradation, not only neutrophil enzymes but also macrophage-derived enzymes such as MMP-12 are important for the development of emphysema-like lesions [27]. A further key enzyme is the macrophage metalloelastase which was reported to mediate acute cigarette smoke-induced inflammation via tumor necrosis factor (TNF)-alpha-release [28]. Neutrophils and macrophages can communicate with other cells such as airway smooth muscle cells, endothelial cells or sensory neurons, and release inflammatory mediators that induce bronchoconstriction [29], airway remodelling [30], and mucin gene induction and mucus hypersecretion involving the induction of mucin genes [31-33].

Lymphocytes are also involved in cellular mechanisms underlying COPD [34,35]. Increased numbers of CD8-positive T-lymphocytes are found in the airways of COPD patients [21,22] and the degree of airflow obstruction is correlated with their numbers [36]. However, the T-cell associated inflammatory processes largely differ from those in allergic asthma, which is characterized by increased numbers of CD4-positive T-lymphocytes [7,37] (Table 2). Although eosinophils may only play a major role in acute exacerbations of COPD [38], their presence in stable disease is an indicator of steroid responsiveness [39-41].

Different inflammatory cell types have also been characterized in airway tissues. Epithelial neutrophilia has been seen in proximal and distal airways of patients with COPD [42,43]. The airway wall beneath the epithelium shows a mononuclear inflammation with increased macrophages and T cells bearing activation markers [20,36] Di Stefano 1996;. An excess od CD8+ T cells are particularly observed in central airways, peripheral airways and parenchyma [20,43]. In the small airways from patients with stage 0 to (at risk) stage 4 (very severe) COPD, the progression of the disease is strongly associated with the accumulation of inflammatory exudates in the small airway lumen and with an increase in the volume of tissue in the airway wall [10]. Also, the percentage of airways containing macrophages, neutrophils, CD4 cells, CD8 cells, B cells, and lymphoid follicle aggregates and the absolute volume of CD8+ T-cells and B cells increased with the progression of COPD [10]. The changes are also most likely associated with an induction of mucin gene expression [44]. The presence of increased numbers of B cells begs the question regarding the role of these cells in the pathophysiology of COPD. In the airway smooth muscle bundles in smokers with COPD, increased localisation of T- cells and neutrophils has been reported, indicating a possible role for these cells interacting with airway smooth muscle in the pathogenesis of airflow limitation [45].

#### Mechanisms of COPD

On the basis of the different pathophysiological mechanisms illustrated in Fig. 1, different animal models have been developed in past years.

##### Protease-antiprotease imbalance

An imbalance between protease and antiprotease enzymes has been hypothesized with respect to the pathogenesis of emphysema [46]. This concept derives from early clinical observations that alpha1-antitrypsin-deficient subjects develop severe emphysema and the role of protease-antiprotease imbalance was later demonstrated in animal models of COPD [47,48]. Although alpha1-antitrypsin-deficiency is a very rare cause of emphysema [49,50], it points to a role of proteases and proteolysis [51,52]. Neutrophil elastase-deficient mice were significantly protected from emphysema-development induced by chronic cigarette smoke [48]. Depletion of the macrophage elastase gene also led to a complete protection from emphysema induced by cigarette smoke [47]. Each of these elastases inactivated the endogenous inhibitor of the other, with macrophage elastase degrading alpha1-antitrypsin and neutrophil elastase degrading tissue inhibitor of metalloproteinase-1 [48]. In tobacco smoke exposure-induced recruitment of neutrophils and monocytes was impaired in elastase gene-depleted animals and there was less macrophage elastase activity due to a decreased macrophage influx in these animals. Thus, a major role for neutrophil elastase and macrophage elastase in the mediation of alveolar destruction in response to cigarette smoke has been shown [47,48]. This experimental evidence derived from animal models points to an important pathogenetic role for proteases that correlates well with the imbalance of proteases present in human COPD. However, many pathways of tissue destruction can be found in animal models that lead to a picture similar to human disease, and it is important to examine whether these mechanisms are operative in the human disease itself.

##### Oxidative stress

Oxidative stress arising from inhaled noxious stimuli such as tobacco smoke or nitrogen dioxide may be important cause of the inflammation and tissue damage in COPD. This potential mechanism is supported by clinical reports of increased levels of oxidative stress indicators in exhaled breath condensates of COPD patients [53-55]. Apart from elevated levels of 8-isoprostane [55], nitrosothiol levels were increased in COPD patients [56-58]. Studies in a mouse model of tobacco smoke-induced COPD also demonstrated the presence of tissue damage due to oxidative stress [59]. These changes could be blocked by superoxide dismutase [60]. Oxidative stress has also been implicated in the development of corticosteroid resistance in COPD.

##### Mediators

Many mediators have been identified which may contribute to COPD pathogenesis [8]. As in bronchial asthma, pro- and anti-inflammatory mediators of inflammation such as tachykinins [61], vasoactive intestinal polypeptide (VIP) [62], histamine [63], nitric oxide [64,65], leukotrienes [66], opioids [67] or intracellular mediators such as SMADs [68,69] have been implicated. The balance of histone acetylases and deacetylases [70] is a key regulator of gene transcription and expression by controlling the access of the transcriptional machinery to bind to regulatory sites on DNA. Acetylation of core histones lead to modification of chromatin structure that affect transcription, and the acetylartion status depends on a balance of histone deacetylase and histone acetyltransferase. This is also likely to play a role in the regulation of cytokine production in COPD. Cigarette smoke exposure led to altered chromatin remodelling with reduced histone deacetylase activity with a resultant increase in transcription of pro-inflammatory genes in lungs of rats exposed to smoke, linked to an increase in phosphorylated p38 MAPK in the lung concomitant with an increased histone 3 phospho-acetylation, histone 4 acetylation and elevated DNA binding of NF-kappaB, and activator protein 1 (AP-1) [70]. In addition, oxidative stress has also been shown to enhance acetylation of histone proteins and decrease histone deacetylase activity leading to modulation of NF-κB activation [71], similar to the effect of cigarette smoke.

A Th2 cytokine that has been proposed to be implicated in the pathophysiology of COPD is IL-13. It is also overexpressed and related to the pathogenesis of the asthmatic Th2 inflammation and airway remodelling process [72]. The effects of IL-13 in asthma have been elucidated in a series of experiments that demonstrated the an airway-specific constitutive overexpression of IL-13 leads to a process of airway remodelling with subepithelial fibrosis and mucus metaplasia combined with an eosinophil-, lymphocyte-, and macrophage-rich inflammation and increased hyperresponsiveness [73]. Since asthma and COPD pathogenesis may be linked, similar mechanisms may contribute to the development and progression of both diseases [74]. In this respect, IL-13 may also play a role in COPD since the inducible overexpression of IL-13 in adult murine lungs leads to alveolar enlargement, lung enlargement and an enhanced compliance and mucus cell metaplasia [75] with activation of MMP-2, -9, -12, -13, and -14 and cathepsins B, S, L, H, and K in this model.

Parallel to protease-based and extracellular mediator-based concepts, altered intracellular pathways may also play a role in COPD. MAPK signalling pathways i.e. p38 and c-Jun N terminal kinase (JNK) [76,77] seem to be important signal transducers in the airways and airway-innervating neurons [78-80] and may therefore display an interesting target for COPD research. For some cells, the activation of p38 or JNK pathways may promote apoptosis rather than proliferation [81,82].

##### Viral infections

Previous studies showed an association between latent adenoviral infection with expression of the adenoviral E1A gene and chronic obstructive pulmonary disease (COPD) [83,84]. It may therefore be assumed that latent adenoviral infection can be one of the factors that might amplify airway inflammation. Human data [35] demonstrating the presence of the viral E1A gene and its expression in the lungs from smokers [85,86], animals [87] and cell cultures [88] support this hypothesis. A small population of lung epithelial cells may carry the adenoviral E1A gene which may then amplify cigarette smoke-induced airway inflammation to generate parenchymal lesions leading to COPD. Inflammatory changes lead to collagen deposition, elastin degradation, and induction of abnormal elastin in COPD [89,90]. Also, latent adenovirus E1A infection of epithelial cells could contribute to airway remodelling in COPD by the viral E1A gene, inducing TGF-beta 1 and CTGF expression and shifting cells towards a more mesenchymal phenotype[84].

#### Genetics

Since only a minority of smokers (approximately 15 to 20%) develop symptoms and COPD is known to cluster in families, a genetic predisposition has been hypothesized. Many candidate genes have been assessed, but the data are often unclear and systematic studies are currently performed to identify disease-associated genes. Next to alpha1-antitrypsin deficiency, several candidate genes have been suggested to be linked to COPD induction. Genetic polymorphisms in matrix metalloproteinase genes MMP1, MMP9 and MMP12 may be important in the development of COPD. In this respect, polymorphisms in the MMP1 and MMP12 genes, but not MMP9, have been suggested to be related to smoking-related lung injury or are in a linkage disequilibrium with other causative polymorphisms [91-93]. An association between an MMP9 polymorphism and the development of smoking-induced pulmonary emphysema was also reported in a population of Japanese smokers [94]. Also, polymorphisms in the genes encoding for IL-11 [95], TGF-beta1 [96], and the group-specific component of serum globulin [97] have been shown to be related to a genetic predisposition for COPD. Since it was difficult to replicate some of these findings among different populations, future studies are needed. Also, whole genome screening in patients and unaffected siblings displays a promising genetic approach to identify genes associated with COPD.

### Experimental models of COPD

**Figure 2:**
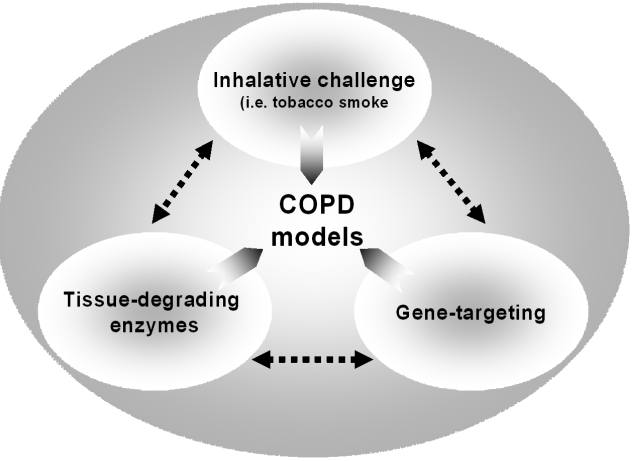
**Experimental approaches to mimic COPD **There are three major experimental approaches to mimic COPD or emphysema consisting of inhalation of noxious stimuli such as tobacco smoke, tracheal instillation of tissue-degrading enzymes to induce emphysema-like lesions and gene-modifying techniques leading to COPD-like murine phenotypes.

There are three major experimental approaches to mimic COPD encompassing inhalation of noxious stimuli, tracheal instillation of tissue-degrading enzymes to induce emphysema-like lesions and gene-modifying techniques leading to a COPD-like phenotype (Figure 2). These approaches may also be combined. Ideally a number of potential indicators for COPD which have been proposed by the GOLD guidelines should be present in animal models of COPD (Table 3). Since COPD definition still rests heavily on lung function measures (airflow limitation and transfer factor), it would be ideal to have lung function measurements in experimental models [15]. The challenge is in the measurement of lung function in very small mammals such as mice and since the use of the enhanced pause (Penh) in conscious mice as an indicator of airflow obstruction is not ideal [98], invasive methods remain the gold standard and these should be correlated with inflammatory markers and cellular remodelling.

**Table 3:** Indicators for COPD. These indicators are related to the presence of COPD and should ideally be present in animal models and available for analysis.

**Indicator**	**Human features**	**Experimental approach**
**History of exposure to risk factors**	Tobacco smoke. Occupational dusts and chemicals. Indoor / outdoor air pollution	Exposure-based experimental protocol
**Airflow obstruction**	Decrease in FEV_1_	Lung function tests
**Hypersecretion**	Chronic sputum production	Functional and morphological assessment of hypersecretion
**Cough**	Chronic intermittent or persistent cough	Cough assessment
**Dyspnea**	Progressive / Persistent / worse on exercise / worse during respiratory infections	Assessment of hypoxemia
**Emphysema**	Progressive impairment of lung function	Morphological analysis of airspace enlargement

#### Inhalation models – tobacco smoke

A variety of animal species has been exposed to tobacco smoke. Next to guinea pigs, rabbits, and dogs, and also rats and mice have been used. Guinea pigs have been reported to be a very susceptible species. They develop COPD-like lesions and emphysema-like airspace enlargement within a few months of active tobacco smoke exposure [99]. By contrast, rat strains seem to be more resistant to the induction of emphysema-like lesions. Susceptibility in mice varies from strain to strain. The mode of exposure to tobacco smoke may be either active via nose-only exposure systems or passive via large whole-body chambers.

The first species to be examined in detail for COPD-like lesions due to tobacco smoke exposure was the guinea pig [99]. Different exposure protocols were screened and exposure to the smoke of 10 cigarettes each day, 5 days per week, for a period of either 1, 3, 6, or 12 months resulted in progressive pulmonary function abnormalities and emphysema-like lesions. The cessation of smoke exposure did not reverse but stabilized emphysema-like airspace enlargement. On the cellular level, long term exposure lead to neutrophilia and accumulation of macrophages and CD4+ T-cells [83,100]. Latent adenoviral infection amplifies the emphysematous lung destruction and increases the inflammatory response produced by cigarette-smoke exposure. Interestingly, it was shown that the increase in CD4+ T-cells is associated with cigarette smoke and the increase in CD8+ T-cells with latent adenoviral infection [83].

Mice represent the most favoured laboratory animal species with regard to immune mechanisms since they offer the opportunity to manipulate gene expression. However, it is more difficult to assess lung function and mice tolerate at least two cigarettes daily for a year with minimal effects on body weight and carboxyhemoglobin levels. Mice differ considerably in respiratory tract functions and anatomy if compared to humans: they are obligate nose breathers, they have lower numbers of cilia, fewer Clara cells and a restriction of submucosal glands to the trachea. Next to a lower filter function for tobacco smoke, mice also do not have a cough reflex and many mediators such as histamine or tachykinins have different pharmacological effects. The development of emphysema-like lesions is strain-dependent: enlarged alveolar spaces and increased alveolar duct area are found after 3–6 months of tobacco smoke exposure in susceptible strains such as B6C3F1 mice [101]. At these later time points, tissue destruction seems to be mediated via macrophages. At the cellular level, neutrophil recruitment has been reported to occur immediately after the beginning of tobacco smoke exposure and is followed by accumulation of macrophages. The early influx of neutrophils is paralleled by a connective tissue breakdown. The early stage alterations of neutrophil influx and increase in elastin and collagen degradation can be prevented by pre-treatment with a neutrophil antibody or alpha1-antitrypsin [102].

Rats are also often used for models of COPD. However, they appear to be relatively resistant to the induction of emphysema-like lesions. Using morphometry and histopathology to assess and compare emphysema development in mice and rats, significant differences were demonstrated [101]: Animals were exposed via whole-body exposure to tobacco smoke at a concentration of 250 mg total particulate matter/m3 for 6 h/day, 5 days/week, for either 7 or 13 months. Morphometry included measurements of tissue loss (volume density of alveolar septa) and parenchymal air space enlargement (alveolar septa mean linear intercept, volume density of alveolar air space). Also, centroacinar intra-alveolar inflammatory cells were assessed to investigate differences in the type of inflammatory responses associated with tobacco smoke exposure. In B6C3F1 mice, many of the morphometric parameters used to assess emphysema-like lesions differed significantly between exposed and non-exposed animals. By contrast, in exposed Fischer-344 rats, only some parameters differed significantly from non-exposed values. The alveolar septa mean linear intercept in both exposed mice and rats was increased at 7 and 13 months, indicating an enlargement of parenchymal air spaces. In contrast, the volume density of alveolar air space was significantly increased only in exposed mice. The volume density of alveolar septa was decreased in mice at both time points indicating damage to the structural integrity of parenchyma. There was no alteration in Fischer-344 rats. Morphologic evidence of tissue destruction in the mice included irregularly-sized and -shaped alveoli and multiple foci of septal discontinuities and isolated septal fragments. The morphometric differences in mice were greater at 13 months than at 7 months, suggesting a progression of the disease. Inflammatory influx within the lungs of exposed mice contained significantly more neutrophils than in rats. These results indicated that B6C3F1 mice are more susceptible than F344-rats to the induction of COPD-like lesions in response to tobacco smoke exposure [101].

Recent work on cigarette exposure in rats indicate that this model also achieves a degree of corticosteroid resistance that has been observed in patients with COPD [103,104]. Thus, the inflammatory response observed after exposure of rats to cigarette smoke for 3 days is noty inhibited by pre-treatment with corticosteroids [70]. This may be due to the reduction in histone deacetylase activity, which could result from a defect in recruitment of this activity by corticosteroid receptors. Corticosteroids recruit hitone deacetylase 2 protein to the transcriptional complex to suppress proinflammatory gene transcription [105]. Modifications in histone deacetylase 2 by oxidative stress or by cigarette smoke may make corticosteroids ineffective [106]. Therefore, models of COPD that show corticosteroid resistance may be necessary and could be used to dissect out the mechanisms of this resistance.

Generally, tobacco smoke exposure may be used to generate COPD features such as emphysema and airway remodelling and chronic inflammation. Although the alterations still differ from the human situation and many involved mediators may have different functional effects especially in the murine respiratory tract, these models represent useful approaches to investigate cellular and molecular mechanisms underlying the development and progression of COPD. As a considerable strain-to-strain and species-to-species variation can be found in the models used so far, the selection of a strain needs to be done with great caution. Animal models of COPD still need to be precisely evaluated as to whether they mimic features of human COPD, and their limitations must be appreciated. Findings obtained from these models may provide significant advances in terms of understanding novel mechanisms involved in COPD.

#### Inhalation models – sulfur dioxide

Sulfur dioxide (SO_2_) is a gaseous irritant which can be used to induce COPD-like lesions in animal models. With daily exposure to high concentrations of SO_2_, chronic injury and repair of epithelial cells can be observed in species such as rat or guinea pig. The exposure to high-levels of this gas ranging from 200 to 700 ppm for 4 to 8 weeks has been demonstrated to lead to neutrophilic inflammation, morphological signs of mucus production and mucus cell metaplasia and damage of ciliated epithelial cells in rats [107,108]. These changes are directly dependent on the exposure to the gas: signs of mucus production and neutrophilic inflammation are almost entirely reversed within a week after termination of exposure [108]. Acute exposure to SO_2 _also leads to loss of cilia and exfoliation of ciliated cells as demonstrated in SO_2_-exposed dogs using transmission electron microscopy [109]. After a longer period of exposure the epithelial layer regenerates and airway wall thickening and change in cilia structure can be observed [110]. Long-term exposure also increases in mucosal permeability both *in vivo *and *in vitro *[111].

Mucus hypersecretion is an important indicator for COPD and experimental models should encompass features of hypersecretion. After chronic exposure to SO_2 _in rats, visible mucus layers and mucus plugs may sometimes be observed in the large airways [107] and an elevation of mucus content may be found in bronchoalveolar lavage fluids [112]. Parallel to these findings, there is an increase of PAS- and Alcian Blue-staining epithelial cells in chronically SO_2 _exposed rats [113] but there is substantial variation present as with human COPD [114]. Tracheal mucus glands are also increased in size after SO_2_-exposure [115] and increased levels of mucin RNA can be found in lung extracts [112]. The mechanisms underlying mucus hypersecretion have not been elucidated so far and also, functional studies assessing basal and metacholine-induced secretion have not been conducted so far.

Airway inflammation with cellular infiltration is an important feature of COPD. After exposure to SO_2_, increases in mononuclear and polymorphonuclear inflammatory cells are present in rat airways. However, the influx is confined to large but not small airways which are important in human COPD [107]. Even after one day of exposure, polymorphonuclear inflammatory cells are found and their influx can be inhibited with steroid treatment [116].

SO_2 _-based models of COPD have also been shown to be associated with an increase in pulmonary resistance and airway hyperresponsiveness [107] and it was hypothesized that elevated levels of mucus may account for the increased responsiveness [117]. Since sensory nerve fibres may function as potent regulators of chronic inflammation in COPD by changes in the activation threshold and the release of pro-inflammatory mediators such as tachykinins [61,118] or CGRP [6,119], this class of nerve fibres was examined in a number of studies [120,121]. The results of these studies supported the hypothesis that rather than contributing to the pathophysiological manifestations of bronchitis, sensory nerve fibres limit the development of airway obstruction and airway hyperresponsiveness during induction of chronic bronchitis by SO_2_-exposure. In this respect, the enhanced contractile responses of airways from neonatally SO_2_-exposed capsaicin-treated rats may result from increased airway smooth muscle mass and contribute to the increased airway responsiveness observed in these animals [121].

To obtain coexisting expression of emphysema and inflammatory changes as seen in COPD, neutrophil elastase instillation and SO_2_-exposure were performed simultaneously [108]. The pre-treatment with elastase aimed to render the animals more susceptible to the inflammation induced by SO_2_. However, neither allergy-phenotype Brown Norway nor emphysematous Sprague–Dawley rats displayed an increased sensitivity to SO_2_-exposure.

With regard to the observed histopathological changes, it can be concluded that SO_2 _exposure leads to a more diffuse alveolar damage with a more extensive damage with destruction of lung tissue after longer exposure. Therefore, the outcome is more or less a picture of tissue destruction with close resemblance to end stages of emphysema but not a complete picture of COPD.

#### Inhalation models – nitrogen dioxide

Nitrogen dioxide (NO_2_) is a another gas that may lead to COPD-like lesions depending on concentration, duration of exposure, and species genetic susceptibility [122]. Concentrations ranging from 50–150 ppm (94–282 mg/m3) can lead to death in laboratory animals due to extensive pulmonary injury including pulmonary oedema, haemorrhage, and pleural effusion.

Short-term exposure to NO2 leads to a biphasic response with an initial injury phase followed by a repair phase. Both increased cellular proliferation and enzymatic activity occur during the repair phase. Exposure of rats to 15 ppm NO_2 _for 7 days leads to an increased oxygen consumption in airway tissues. The increase in oxidative capacity reflects an increase in mitochondrial activity consistent with observations of increased DNA synthesis [123]. Exposure to 10 ppm NO_2 _for more than 24 h causes damage to cilia and hypertrophy of the bronchiolar epithelium [124]. Also, exposure to 15–20 ppm NO_2 _leads to a type II pneumocyte hyperplasia [125,126].

As with the exposure to other noxious stimuli, there is also a significant inter-species variability. In comparison to mice and rats, guinea pigs exhibit changes in lung morphology at much lower NO_2 _concentrations. It was shown that a 2 ppm NO_2 _3-day exposure causes increased thickening of the alveolar wall, damage to cilia and pulmonary oedema [127]. Other changes are an influx of inflammatory cells and increases in connective tissue formation [128].

There is also a significant mode of inheritance of susceptibility to NO_2_-induced lung injury in inbred mice. Susceptible C57BL/6J (B6) and resistant C3H/HeJ (C3) mice, as well as F1, F2, and backcross (BX) populations derived from them, were acutely exposed to 15 parts per million NO_2 _for 3 h to determine differences [122]. Significant differences in numbers of lavageable macrophages, epithelial cells, and dead cells were found between inbred strains: distributions of cellular responses in F1 progeny overlapped both progenitors, and mean responses were intermediate. It was shown that in C3:BX progeny, ranges of responses to NO_2 _closely resembled C3 mice. Ranges of cellular responses to NO_2 _in B6:BX and intercross progeny were reported to overlap both progenitor and mean responses of both populations were intermediate to progenitors. Therefore, there were likely two major unlinked genes that account for differential susceptibility to acute NO_2 _exposure [122]. Based on the genetic background of C57BL/6 mice, a model of long-term NO_2 _exposure was recently established leading to signs of pulmonary inflammation and progressive development of airflow obstruction [129].

#### Inhalation models – oxidant stimuli and particulates

The administration of oxidants such as ozone also causes significant lung injury with some features related to inflammatory changes occurring in human COPD [130] and this causes numerous effects in airway cells [131-135]. As a gaseous pollutant, ozone targets airway tissues and breathing slightly elevated concentrations of this gas leads to a range of respiratory symptoms including decreased lung function and increased airway hyper-reactivity. In conditions such as COPD and asthma, ozone may lead to exacerbations of symptoms. Ozone is highly reactive: the reaction with other substrates in the airway lining fluid such as proteins or lipids leads to secondary oxidation products which transmit the toxic signals to the underlying pulmonary epithelium. These signals include cytokine generation, adhesion molecule expression and tight junction modification leading to inflammatory cell influx and increase of lung permeability with oedema formation [130]. However, the nature and extent of these responses are often variable and not related within an individual. The large amount of data obtained from animal models of ozone exposure indicates that both ozone- and endotoxin-induced animal models are dependent on neutrophilic inflammation. It was shown that each toxin enhances reactions induced by the other toxin. The synergistic effects elicited by coexposure to ozone and endotoxin are also mediated, in part, by neutrophils. [136,137].

Further animal models focus on the exposure to ultrafine particles, silica and coal dust [138,139]. Ultrafine particles are a common component of air pollution, derived mainly from primary combustion sources that cause significant levels of oxidative stress in airway cells [140,141]. The animal models are predominantly characterized by focal emphysema and it was suggested that dust-induced emphysema and smoke-induced emphysema occur through similar mechanisms [142].

Exposure to diesel exhaust particles (DEP) may also lead to chronic airway inflammation in laboratory animals as it was shown to have affect various respiratory conditions including exacerbations of COPD, asthma, and respiratory tract infections [143]. Both the organic and the particulate components of DEP cause significant oxidant injury and especially the particulate component of DEP is reported to induce alveolar epithelial damage, alter thiol levels in alveolar macrophages (AM) and lymphocytes, and induce the generation of reactive oxygen species (ROS) and pro-inflammatory cytokines [144]. The organic component has also been shown to generate intracellular ROS, leading to a variety of cellular responses including apoptosis. Long-term exposure to various particles including DEP, carbon black (CB), and washed DEP devoid of the organic content, have been shown to produce chronic inflammatory changes and tumorigenic responses [144]. The organic component of DEP also suppresses the production of pro-inflammatory cytokines by macrophages and the development of Th1 cell-mediated mechanisms thereby enhancing allergic sensitization. The underlying mechanisms have not been fully investigated so far but may involve the induction of haeme oxygenases, which are mediators of airway inflammation [145]. Whereas the organic component that induces IL-4 and IL-10 production may skew the immunity toward Th2 response, the particulate component may stimulate both the Th1 and Th2 responses [146]. In conclusion, exposure to particulate and organic components of DEP may be a helpful approach to simulate certain conditions such as exacerbations. Also, the development of lung tumours after long term exposure may be useful when studying interactions between COPD-like lesions and tumorigenesis.

A further toxin is cadmium chloride, a constituent of cigarette smoke. Administration of this substance also leads to alterations in pulmonary integrity with primarily interstitial fibrosis with tethering open of airspaces [147]. A combination of cadmium and lathyrogen beta-aminopropionile enhances emphysematous changes [148].

#### Tissue-degrading approaches

Emphysema-like lesions can also be achieved by intrapulmonary challenge with tissue-degrading enzymes and other compounds [149] (Figure 2). Proteinases such as human neutrophil elastase, porcine pancreatic elastase, or papain produce an efficient enzymatic induction of panacinar emphysema after a single intrapulmonary challenge [150,151]. Since bacterial collagenases do not lead to the formation of emphysema, the effectiveness of the proteinases is related to their elastolytic activity. While these models may not be as useful as smoke exposure studies to achieve COPD-like lesions, they can lead to a dramatic picture of emphysema and may be used to study mechanisms related specifically to emphysema and to the repair of damaged lung. However, the method of inducing emphysema-like lesions by intratracheal instillation of these enzymes may not very closely relate to mechanisms found in the human situation.

Among the different emphysema models, elastase-induced emphysema has also been characterized to be accompanied by pulmonary function abnormalities, hypoxemia, and secretory cell metaplasia which represent characteristic features of human COPD. Recent studies suggested that exogenous retinoic acid can induce alveolar regeneration in models of elastase-induced experimental emphysema [152] and that retinoic acid may have a role for alveolar development and regeneration after injury [153,154]. However, the role of retinoic acid in relation to alveolar development has only been analysed in a rat model and models in other animals did not show similar effects [155]. Also, the ability of alveolar regeneration which is present in rats does not occur to a similar extent in humans; a recent clinical trial using retinoic acid in COPD did not show positive results [156].

The mechanisms of emphysema induction by intratracheal administration of elastase encompass an initial loss of collagen and elastin. Later, glycosaminoglycan and elastin levels normalize again but collagen levels are enhanced. The extracellular matrix remains distorted in structure and diminished with resulting abnormal airway architecture [157]. The enlargement of the airspaces immediately develops after the induction of elastolytic injuries and is followed by inflammatory processes which lead to a transformation of airspace enlargement to emphysema-like lesions. This progression most likely occurs due to destructive effects exerted by host inflammatory proteinases. Addition of lathyrogen beta-aminopropionile leads to an impairment of collagen and elastin crosslinking and therefore further increases the extent of emphysema-like lesions [158]. Effects seem to be mediated via IL-1β and TNFα receptors since mice deficient in IL-1β Type1 receptor and in TNFalpha type 1 and 2 receptors are protected from developing emphysema following intratracheal challenge with porcine pancreatic elastase. This was associated with reduced inflammation and increased apoptosis [159].

In general, intrapulmonary administration of tissue-degrading enzymes represents a useful tool especially when focusing on mechanisms to repair emphysematic features. However, the lack of proximity to the human situation needs to be realized since the mechanisms of emphysema induction are clearly not related to the human situation. An advantage of proteinase-based models is the simple exposure protocol with a single intratracheal administration leading to significant and rapid changes. However, extrapolating these findings to slowly developing features of smoking induced human COPD is very difficult since a large number of mediators may not be involved in the rapid proteinase approach. Therefore, these models may not encompass important features of human COPD which may be more closely mimicked by inhalation exposures and it is clear that tissue-degrading enzyme models always represent the picture of an "induced pathogenesis".

#### Gene-targeting approaches

The genetic predisposition to environmental disease is an important area of research and a number of animal strains prone to develop COPD-like lesions have been characterized [160-162] (Figure 2). Also, genetically-altered monogenic and polygenic models to mimic COPD have been developed in recent years using modern techniques of molecular biology [163,164].

Gene-depletion and -overexpression in mice provide a powerful technique to identify the function and role of distinct genes in the regulation of pulmonary homeostasis *in vivo*. There are two major concepts consisting of gain-of-function and loss-of-function models. Gain-of-function is achieved by gene overexpression in transgenic mice either organ specific or non-specific while loss of function is achieved by targeted mutagenesis techniques [165,166]. These models can be of significant help for the identification of both physiological functions of distinct genes as well as mechanisms of diseases such as COPD.

A large number of genetically-altered mice strains have been associated to features of COPD and a primary focus was the assessment of matrix-related genes. As destruction of alveolar elastic fibres is implicated in the pathogenic mechanism of emphysema and elastin is a major component of the extracellular matrix, mice lacking elastin were generated. It was shown that these animals have a developmental arrest development of terminal airway branches accompanied by fewer distal air sacs that are dilated with attenuated tissue septae. These emphysema-like alterations suggest that in addition to its role in the structure and function of the mature lung, elastin is essential for pulmonary development and is important for terminal airway branching [167]. Also, deficiency of the microfibrillar component fibulin-5 and platelet derived growth factor A (PDGF-A) leads to airspace enlargement [168,169]. PDGF-A(-/-) mice lack lung alveolar smooth muscle cells, exhibit reduced deposition of elastin fibres in the lung parenchyma, and develop lung emphysema due to a complete failure of alveogenesis [170]. The postnatal alveogenesis failure in PDGF-A(-/-) mice is most likely due to a prenatal block in the distal spreading of PDGF-R alpha+ cells along the tubular lung epithelium during the canalicular stage of lung development [170].

The importance of integrins in causing emphysema has been demonstrated in mouse. Epithelial restricted integrin α vβ 6-null mice develop age-related emphysema through the loss of activation of latent TGF-beta which leads to an increase in macrophage MMP-12 expression [171].

Fibroblast growth factors are known to be essential for lung development. Mice simultaneously lacking receptors for FGFR-3 and FGFR-4 have an impaired alveogenesis with increased collagen synthesis [172]. It is crucial to distinguish developmental airspace enlargement from adult emphysema which is defined as the destruction of mature alveoli. However, the identification of numerous factors influencing lung development is an important step towards identifying potential mechanisms underlying the development and progression of emphysema in human COPD.

Next to developmental airspace enlargement also spontaneous emphysema may occur in genetically-modified mice strains and a gradual appearance of emphysema-like lesions has been found in mice lacking the surfactant protein D (SP-D) gene [173] and in mice lacking the tissue inhibitor of metalloproteinase-3 (TIMP-3) gene [174]. In these strains, matrix metalloproteinases were suggested to be the primary mediators of tissue destruction.

A further mechanism to induce emphysema-like lesions is to expose developmentally normal genetically-modified animals to exogenous noxious stimuli such as tobacco smoke. This also allows identifying potential molecular mechanisms involved in the pathogenesis of COPD. Using macrophage elastase (MMP-12) gene-depletion studies it was shown that in contrast to wild type mice, the lung structure of MMP-12 gene-depleted animals remains normal after long term exposure to cigarette smoke [47]. These animals also fail to develop macrophage accumulation in response to cigarette smoke, an effect that could be related to MMP-12 induced generation of elastin fragments that are chemotactic for monocytes [175,176].

In summary, gene-targeting techniques display very useful tools to examine potential molecular mechanisms underlying human COPD. In combination with inhalation protocols they may identify important protective or pro-inflammatory mediators of the disease.

#### Other models

Various other agents have also been characterized to induce airway inflammation injury. In this respect, administration of toxins such as endotoxin leads to a recruitment of neutrophils and macrophage activation with concomitant airspace enlargement [177,178].

Non-inflammatory emphysema-like lesions may also be accomplished by intravascular administration of a vascular endothelial cell growth factor receptor-2 (VEGFR-2) blocker [179]. VEGF is required for blood vessel development and endothelial cell survival and its absence leads to endothelial cell apoptosis [180]. An increased septal cell death in human emphysematous lungs and a reduced expression of VEGF and VEGFR-2 is found in emphysema lungs [181]. Also, chronic blockage of VEGFR-2 causes alveolar septal cell apoptosis and airspace enlargement [179]. These findings of airspace enlargement point to a role of the vascular system in the development and progression of emphysema.

## Conclusions

In contrast to the variable pathology and different stages of severity in human COPD, currently available animal models are restricted to mimicking a limited amount of characteristic features of COPD. Animal models need to be precisely evaluated based on whether they agree with features of human COPD in order to advance the understanding of mechanisms in human COPD.

Based on inhalative exposure to noxious stimuli such as cigarette smoke, the administration of tissue-degrading enzymes or gene-targeting techniques, a number of experimental approaches to mimic acute and chronic features of COPD have been established in the past years. Due to the complexity of the disease, and species-specific differences they are all limited concerning their clinical significance.

While the induction of the COPD lesions by tissue-degrading enzymes may appear artificial in many cases, it does not mean that these models are not valuable because they can be used to study many aspects of pulmonary pathophysiology of end-stage emphysema. Cellular mechanisms can be studied efficiently and underlying molecular mechanisms and potential therapeutic approaches can be revealed if the data is extrapolated cautiously.

Combined models of inhalative exposure, proteinase-based tissue degradation to produce emphysema and gene-targeting techniques may provide models of COPD which encompass more features of the disease. However, one cannot assume that reproducing COPD with a high degree of fidelity in the animal necessarily means that the model simulates the human condition. In fact, a model that only produces a single pathologic COPD feature may be more useful as long as it produces this feature via a relevant mechanism that allows exploratory research. By contrast, a model producing all kinds of COPD features via irrelevant mechanisms may be less useful. In this respect, validation of models as being relevant is an extremely important issue in the early steps of model development. Animal models should not only assess histopathological features but also attempt to focus on functional features of human COPD such as airflow limitation, mucus hypersecretion, chronic cough and exacerbations, and also on pharmacological features such as corticosteroid resistance or diminished β-adrenergic bronchodilator responses. In conclusion, there are many benefits that can accrue from the development of animal models of COPD, most important of which is understanding of mechanisms and development of specific drugs for COPD.
